# The Relationships Between Common Measurements of Taste Function

**DOI:** 10.1007/s12078-015-9183-x

**Published:** 2015-06-05

**Authors:** Jordannah Webb, Dieuwerke P. Bolhuis, Sara Cicerale, John E. Hayes, Russell Keast

**Affiliations:** Centre for Advanced Sensory Science, School of Exercise and Nutrition Sciences, Deakin University, 221 Burwood Highway, Burwood, Victoria 3125 Australia; Sensory Evaluation Center, College of Agricultural Sciences, Pennsylvania State University, State College, PA USA

**Keywords:** Taste function, Suprathreshold, Threshold, Fungiform papillae, PROP

## Abstract

**Background:**

There are five common, independent measures used to characterize taste function in humans: detection and recognition thresholds (DT and RT), suprathreshold intensity ratings of prototypical tastants, propylthiouracil (PROP) bitterness intensity, and fungiform papillae (FP) number.

**Methods:**

We employed all five methods to assess taste function of 65 women (21.5 ± 4 years, BMI 22.3 ± 2.8 kg/m^2^). Pearson correlation coefficients were calculated between the different measures.

**Results:**

The DT and RT were positively correlated for sweet, bitter, sour, and umami (*p* < 0.05), but not for salt. The DT or RT did not correlate with suprathreshold intensity ratings, except for umami (suprathreshold intensity and RT: *r* = −0.32, *p* = 0.009). FP number did not correlate with any measurement of taste function. PROP bitterness intensity ratings did not correlate with any measurement of taste function, except for suprathreshold ratings for saltiness (*r* = 0.26, *p* = 0.033).

**Conclusion:**

As most of the individual measures of taste function did not correlate with each other, with exception of the two threshold measures, we conclude that there are multiple perceptual phases of taste, with no single measure able to represent the sense of taste globally.

## Introduction

The sense of taste is the gatekeeper to ingestion. From an evolutionary perspective, taste played a critical role in the survival of species by informing about nutrients or toxins in potential foods (Cordain et al. 2005). In the modern environment, our sense of taste does not play such a central role for survival but may still have a significant role in dietary choice (Connors et al. 2001; Duffy, 2004; Glanz et al. 1998). Taste sensors are located in papillae throughout the oral cavity. These papillae house taste receptor cells (TRCs), which are stimulated when nonvolatile chemicals enter the mouth. TRCs synapse onto afferent fibers that project to cortical regions of the brain; with sufficient stimulation, the impulse is decoded, and we perceive a taste (Bachmanov and Beauchamp 2007). There is large interindividual variation in taste perception (see review by Hayes et al. 2013), where such variation may arise from differences in human physiology or the cognitive processing of taste signals. There is no single method to assess global taste function; indeed, five distinct methods are commonly employed by researchers studying chemosensation or ingestive behavior. Detection and recognition thresholds provide estimates of the lowest chemical concentration that can be perceived by an individual. For example, a solution may contain a substance at a concentration undetectable to the general population, but as the concentration is increased, a detection threshold (DT) is attained such that the solution can be discriminated from pure solvent in a forced choice task. As the concentration is further increased, a recognition threshold (RT) is attained, and this is the point where the substance is both perceived and identifiable as having a specific perceptual quality (Keast and Roper 2007).

It is widely accepted that individuals with lower DT and RT are more sensitive to a particular chemical than those with a higher DT and RT. In contrast, suprathreshold intensity refers to the perceived intensity (magnitude) of a substance at concentration above threshold. As the stimulus concentration increases, it is expected that the perceived intensity will also increase, eventually reaching to a terminal threshold for the stimulus and quality. Suprathreshold intensity of prototypical tastants is a third means to quantify taste function. These tests occur at concentrations between the recognition threshold and the terminal threshold, and intensity of the same stimulus can vary widely across individuals (e.g., Allen et al. 2013). The fourth measure often used is propylthiouracil (PROP) bitterness; PROP is extremely bitter for some, while others will perceive little or no bitterness (Bartoshuk et al. 1994; Tepper et al. 2009). PROP has been previously used to identify individuals, known as supertasters, who find this chemical to be intensely bitter. Subsequently, this term has also been applied to individuals who show heightened taste response across multiple qualities, not just PROP bitterness (Hayes et al. 2008). More recently, it was suggested that the terminology supertaster can be confusing, as it may refer to a narrow trait relating to PROP, or the broad trait of heightened taste response to a broad range of stimuli (Hayes and Keast 2011). The fifth measure commonly used is the quantification of fungiform papillae (FP) anatomy. FP contain taste buds, and it is due to their abundance and location on the anterior tongue that they are chosen for quantification when compared to foliate and circumvallate papillae (Huguley 1990). FP are small, mushroom-shaped structures, and the densities of these structures have been shown to vary largely between individuals. Presumably, the more FP an individual has, a stronger signal is sent centrally from the FP, resulting in a more intense taste perception (Essick et al. 2003; Miller and Reedy 1990; Zhang et al. 2009; Hayes and Duffy 2007; Hayes et al. 2010), although not all studies support this contention (Garneau et al. 2014; Feeney and Hayes 2014; Fischer et al. 2013).

The aim of the present study is to investigate how these five distinct measures commonly used to assess taste function relate to each other.

## Materials and Methods

### Study Design

This study comprised five methods of taste assessment routinely used in chemosensory research measured over two sessions on separate days: (1) detection threshold (DT), (2) recognition threshold (RT), (3) suprathreshold intensity of the five prototypical tastes, (4) suprathreshold bitterness of propylthiouracil (PROP), and (5) fungiform papillae quantification. Demographic information was also collected, including gender, age, height, and weight. Body mass index (BMI, kg/m^2^) was calculated from the height and weight measurements. DT, RT, and suprathreshold intensity procedures were conducted in computerized, partitioned sensory booths in the Centre for Advanced Sensory Science using Compusense Five Software Version 5.2 (Compusense Inc., Ontario, Canada). Fungiform papillae photography (Haryono et al. 2014) and PROP testing (Zhao et al. 2003) were also conducted within this laboratory using standard methods.

### Subjects

The subjects (*n* = 65, 21.5 ± 4 years) were female university students, between the ages of 18 and 42. The subjects were asked to refrain from eating, drinking (except room temperature water), brushing teeth, or chewing gum for 1 h prior to testing.

### Subject Training

Subjects were trained in the use of the general labeled magnitude scale (gLMS) following published standard procedures (Bartoshuk 2000; Green et al. 1993, 1996) that involved culturally appropriate remembered or imagined sensations, such as the the coolness of an ice-cold beverage, or the sweetness of fairy floss (known as candy floss in the UK, or cotton candy in the USA). The gLMS is a psychophysical tool that yields high quality, ratio level data (Bartoshuk 2000). It requires subjects to rate their perceived intensity of a given stimulus along a line scale with adjectives at empirically derived intervals. The 100 point scale comprises the following adjectives: no sensation = 0, barely detectable = 1.5, weak = 6, moderate = 17, strong = 35, very strong = 52, and the strongest imaginable sensation of any kind = 100 (Bartoshuk 2000). The scale presented to subjects shows only the adjectives, not the corresponding numbers; however, when collating results, the experimenter extrapolates numerical data from the scale.

### Stimuli

The chemicals used to make the taste solutions included the following: sucrose (sweet) (CSR, Yarraville, Australia); sodium chloride (NaCl) (salty) (Saxa, Premier Foods Inc, UK); citric acid (sour) (IMCD group, New Zealand); caffeine and PROP (bitter) (Sigma Aldrich, Steinham, Germany); and monosodium glutamate (MSG) (umami) (Fuzhou Golden Banyan Foodstuffs, China). All solutions were prepared in accordance with the International Standards Organisation (ISO3972 1991). On the morning of testing, solutions were prepared with filtered deionized water and were stored in glass beakers at room temperature. Filtered deionized water was used as an oral rinsing agent for taste threshold and suprathreshold intensity experiments. All samples for threshold and suprathreshold intensity testing were served in 15-ml portions at room temperature, with a three digit blinding code allocated to each sample. Preparation of PROP filter paper is detailed below.

### Detection and Recognition Threshold Determination for the Five Primary Tastes

To determine DT and RT, a modified testing method was developed using the procedure outlined in the ISO standards (ISO3972 1991). Detailed in Table 1 are the ten chemical concentrations used for each taste quality, where the first eight concentrations were presented to subjects. The ninth concentration was presented when subjects were unable to perceive any taste from the previous eight concentrations, and the tenth concentration was used when no taste was perceived from any of the previous concentrations. The eight samples from each taste quality were served in ascending concentration (in accordance with the standard ISO method), and each taste quality was presented to subjects independently. Subjects were unaware of the presentation order but were informed of the possible taste qualities. Subjects were instructed to taste each sample for 5 s then spit and record whether there was an absence of taste (water-like), taste identified but quality unknown, or taste quality perceived. DT was defined as the concentration at which the response “taste identified but quality unknown” was selected. RT was defined as the concentration at which the taste quality was correctly identified twice consecutively.

**Table 1:** Concentrations of the taste solutions used for threshold testing (mM)

Taste quality	Reference chemical	Sample concentrations (mM)									
		1	2	3	4	5	6	7	8	9	10
Sweet	Sucrose	1.0	1.6	2.7	4.5	7.5	12.6	21.0	35.0	70.0	140
Salty	Sodium chloride	2.7	4.1	5.8	8.2	11.8	16.8	24.0	34.2	68.4	137
Sour	Citric acid	0.7	0.8	1.0	1.3	1.6	2.0	2.5	3.1	6.2	12.4
Bitter	Caffeine	0.3	0.4	0.5	0.6	0.7	0.9	1.1	1.4	2.8	5.6
Umami	MSG	0.5	0.7	1.0	1.4	2.0	2.9	4.1	5.9	11.8	23.6

### Suprathreshold Intensity Ratings of the Five Prototypical Tastes

For each prototypical tastant, three concentrations (low, medium, and high) were prepared to determine perceived suprathreshold intensity (Table 2). The three concentrations were served in a randomized order, and each tastant was presented to subjects independently. Subjects were instructed to taste each sample and record their perceived overall intensity on a computerized gLMS.

**Table 2:** Concentrations of the taste solutions used for suprathreshold intensity testing (mM)

Taste quality	Reference substance	Sample concentrations (mM)		
		Weak	Medium	Strong
Sweet	Sucrose	100	200	400
Salty	Sodium chloride	100	200	400
Sour	Citric acid	1.0	3.0	7.0
Bitter	Caffeine	1.0	2.0	4.0
Umami	MSG	3.0	6.0	12.0

### Standardization of gLMS Usage with Weight Ratings

To control for idiosyncratic scale usage, subjects were asked to rate the heaviness of six visually identical weights (sand-filled opaque jars of weights 53, 251, 499, 724, 897, and 1127 g). Subjects held out their dominant hand, palm up while the experimenter placed the weighted bottle on the palm of the hand. Subjects were instructed to use the gLMS to rate the heaviness of each weight. Significant correlations were found between PROP bitterness and average heaviness of the weights rated using the gLMS (*r* = 0.46, *p* < 0.01). Given that these two variables should be unrelated, this indicates that the ratings made using a gLMS were subject to idiosyncratic scale usage, and following published standard procedures, the mean heaviness of the weights was used as a means of standardizing the ratings made using the gLMS (Keast et al. 2003).

### Quantification of Fungiform Papillae

Preparation for FP photography was carried out using a 10 × 2.5-cm piece of filter paper to dry and remove excess saliva from the front section of the tongue. A cotton tip was then immersed in a diluted (1:5, dye/water) solution of dye (2.1 % blue dye, Queen Fine Foods, Australia) (Haryono et al. 2014). The area to be stained blue was left of the tongue midline on the anterior region (between 0 and 3 cm from the tip), as this area has been used previously (Essick et al. 2003; Miller and Reedy 1990; Tepper and Nurse 1997; Yackinous and Guinard 2002) and was determined to be highly correlated with the total number of FP on the tongue (Shahbake et al. 2005). Following dye application, the tongue was dried once more with a 10 × 2.5-cm piece of filter paper removing excess dye and revealing the lighter stained FP. A 1 × 1.5-cm piece of paper containing a circle cut out 6-mm diameter was then placed over the dyed section of the tongue. The 6-mm diameter area has been shown to be a reliable measure of FP density on the anterior tongue (Shahbake et al. 2005). The camera (Nikon D90 DSLR and 105 mm f*/*2.8G lens) was stabilized with use of a tripod, and photos were taken in triplicate. The clearest shot from each photoset was chosen for papillae counting, and counts were made in Adobe Photoshop (Adobe Systems Incorporated: Version 12).

### PROP Bitterness

A 50-mM/L PROP solution was made by dissolving PROP powder (8.5 g) in 1 L of boiling water (100 °C) on a stirring hotplate. Large sheets of filter paper (30 × 40 cm) were soaked in the PROP solution for 30 s. Excess solution was removed and the filter paper dried in a 120 °C fan-forced oven (duration approximately 30 min). The dried filter paper was then cut into 2.5 × 2.0-cm pieces and presented to the subjects who were instructed to place the paper on the center of their tongues. The gLMS was used to rate the bitter intensity of the PROP paper.

### Statistical Analysis

Statistical analysis was performed using IBM SPSS statistical software version 22 (SPSS Inc, Chicago, IL, USA). Data are presented as means with standard errors (SE). A Pearson’s product moment correlation coefficient was calculated between distinct measures of taste function. The threshold and suprathreshold ratings were log-transformed before correlations were assessed. For suprathreshold intensity ratings, the geometric mean of the three ratings (weak, medium, and strong) was calculated. Student’s *t*-test for independent groups was used to assess differences between the subjects in the top quintile group that rated PROP as most intensely bitter versus the remaining subjects. The criterion for statistical significance was set at alpha = 0.05.

## Results

### Detection and Recognition Thresholds

DT and RT of the five prototypical tastes are presented in Table 3. A strong correlation between DT and RT was observed for bitter (*r* = 0.81, *p* < 0.001), and correlations were observed for sweet (*r* = 0.32, *p* = 0.009), and umami (*r* = 0.50, *p* < 0.001), and sour (r = 0.24, *p* = 0.05); conversely, DT and RT were not correlated for the salty stimulus (*r* = 0.17, *p* = 0.18).

**Table 3:** Taste thresholds (mM) presented as mean and standard error

	Detection threshold		Recognition threshold	
	Mean	SE	Mean	SE
Sucrose	10.9	1.48	29.3	3.08
Sodium chloride	4.97	0.37	37.0	4.52
Citric acid	0.70	0.002	0.90	0.09
Caffeine	0.78	0.14	1.10	0.15
MSG	1.38	0.12	4.11	0.55

DT of sweet, salty, sour, and umami were positively correlated with each other (*r* = 0.3–0.4, all *p* values <0.01). However, DT of sour was not correlated with the other taste qualities. RT of sweet and sour were positively correlated (*r* = 0.26, *p* = 0.04), umami and bitter RTs were positively correlated (*r* = 0.29, *p* = 0.02), and there was a marginal relationship for umami and sour RTs (*r* = 0.24, *p* = 0.05).

### Suprathreshold Intensities and Relationship with Detection and Recognition Threshold

As expected, there were monotonic increases in perceived intensity as the concentration of stimuli was increased (Table 4). The geometric mean of the suprathreshold intensities was strongly correlated between all five taste qualities (*r* = 0.34 to 0.56, all *p* values <0.007), even after ratings were standardized with weight, consistent with the ideal of generalized hypergeusia (Hayes and Keast 2011).

**Table 4:** Suprathreshold intensity concentrations presented as geometric mean (GM) and standard error (SE)

		GM	SE
Sucrose	100 mM	6.95	0.90
	200 mM	12.6	1.25
	400 mM	19.1	1.69
Sodium chloride	100 mM	7.9	1.24
	200 mM	18.0	1.45
	400 mM	24.0	1.73
Citric acid	1.0 mM	5.40	0.80
	3.0 mM	13.2	1.30
	7.0 mM	18.7	1.47
Caffeine	1.0 mM	10.6	1.57
	2.0 mM	18.7	2.16
	4.0 mM	26.6	2.48
MSG	3.0 mM	2.61	0.66
	6.0 mM	4.34	0.89
	12.0 mM	7.24	1.14

**Fig. 1:**
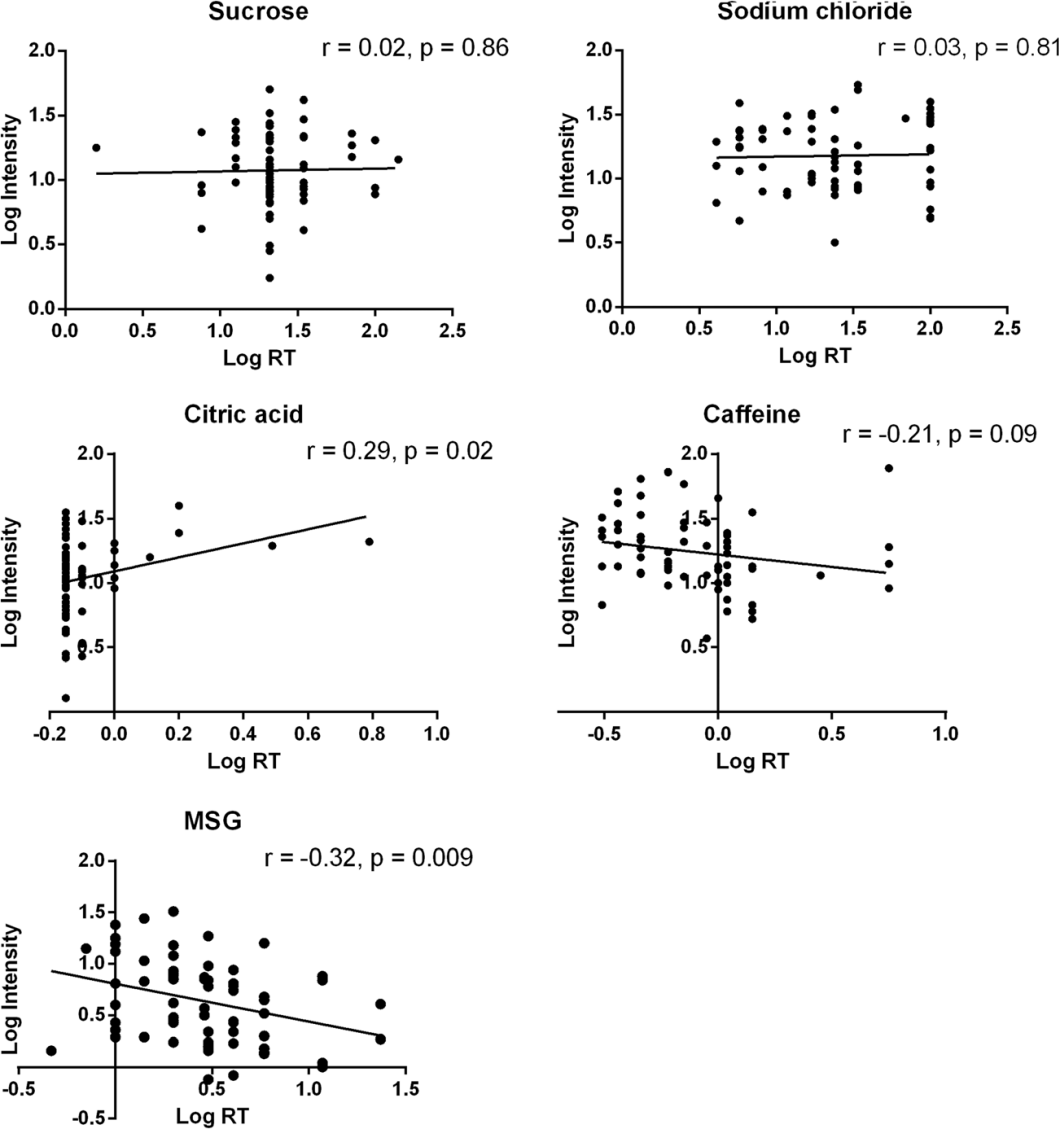
Scatter plots and correlation coefficients of log RT and log suprathreshold intensity for each taste quality. For suprathreshold intensity ratings, the geometric mean of the three ratings (weak, medium, and strong) was used

No correlations were observed between suprathreshold intensities and DT of any taste qualities (all *p* values >0.05). Figure 1 shows scatter plots and correlations between RTs and suprathreshold intensity ratings for each taste quality. A negative correlation was observed for umami. A trend for negative correlation for bitter was observed. RT and suprathreshold intensity for sour were positively correlated. No correlations were observed for sweet and salt.

### PROP Intensity Ratings and Relationships with Other Measurements of Taste Function

Large variation in perceived PROP bitterness was noted with a minimum rating of 3.5, maximum of 56.2, and a mean of 23.5 ± 1.60. PROP intensity ratings were positively correlated with suprathreshold intensity for saltiness (*r* = 0.26, *p* = 0.033), and a trend was observed for suprathreshold intensity for sourness (*r* = 0.24, *p* = 0.06). However, PROP intensity ratings were not correlated with the other measures of taste response.

**Fig. 2:**
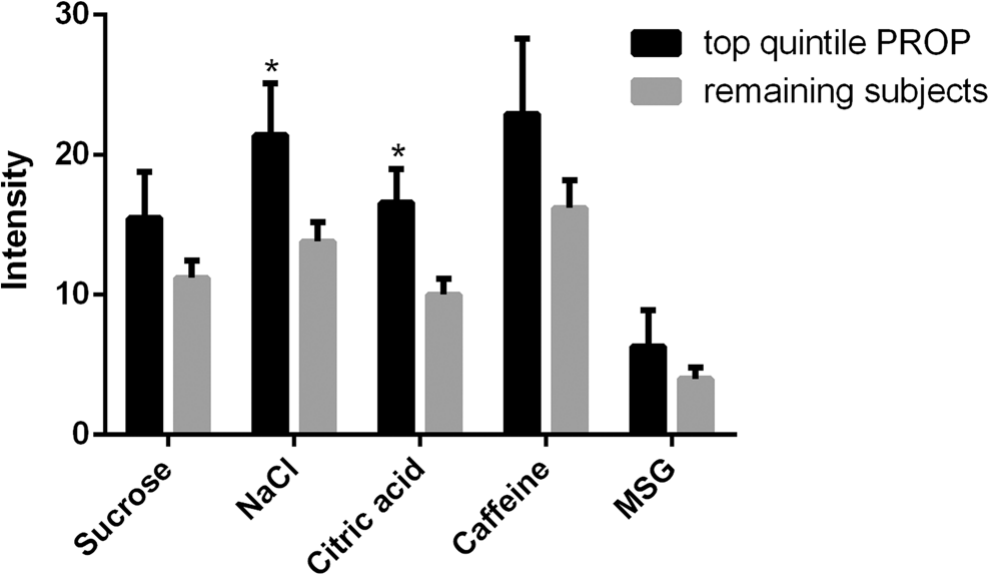
Taste intensity rating of the five primary tastes. The top quartile subjects who tasted PROP as most bitter (*n* = 13) versus the remaining subjects (*n* = 52). Sodium chloride and citric acid were rated as more intense by the subjects of the top quartile (*p < 0.05). Sucrose, caffeine, and MSG tended to be rated as more intense by the subjects in the top quartile (*p* < 0.1)

Moreover, we observed that those who rated PROP as most bitter (top quintile, *n* = 13) were also more sensitive to the five prototypical tastes, with a higher average suprathreshold intensity rating (all five tastants), t(65) = −2.64, *p* = 0.01, consistent with prior data (Hayes et al. 2008). Figure 2 shows the differences in intensity ratings for all tastants separately for the top quintile versus the remaining subjects. Subjects in the top quintile did not have different DT or RT in any of the taste qualities compared to the remaining subjects (all *p* values >0.05).

### Fungiform Papillae Number and Relationships with Other Measurements of Taste Function

**Fig. 3:**
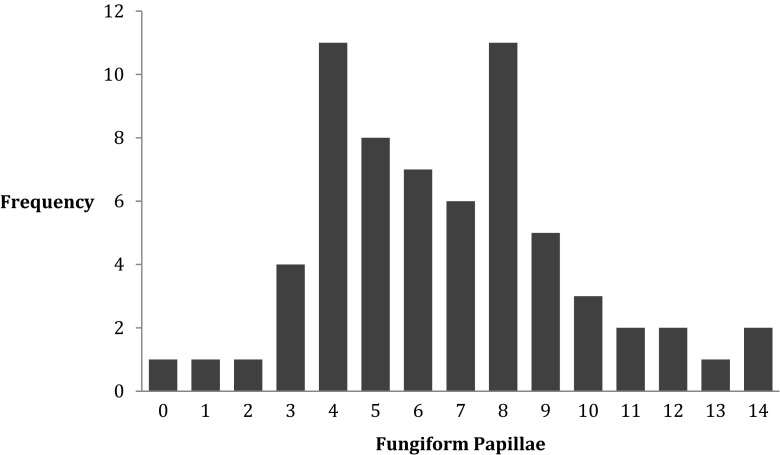
Frequency distribution of fungiform papillae density (per 28.3 mm^2^)

FP number followed a normal distribution (Fig. 3), where the mean FP number (±SD) for the 6-mm stained region of the tongue (area 28.3 mm^2^) was 6.66 ± 3.01. A minimum of 0 and a maximum of 14 FP were observed. None of the measures of taste function were correlated with FP number. The strength of the correlations between FP number and DT ranged from *r* = ±0.03–0.10, all *p* values >0.05. The correlations between FP number and RT ranged from *r* = ±0.02–0.15, all *p* values >0.05, between FP density and suprathreshold intensities *r* = −0.02–−0.24, all *p* values >0.05. FP density and PROP bitterness did not show a correlation (*r* = −0.08, *p* = 0.5).

## Discussion

These results suggest that taste function is extremely complex and difficult to characterize, given that the five common ways to identify taste functioning were shown to be mostly unrelated. Only DT and RT were related; however, these threshold measurements poorly correlated with suprathreshold intensity, and not with number of FP or PROP bitterness intensity. This also suggests that each individual measure characterizes a separate component of the sense of taste, and no single measure is capable of being used as a definitive marker of overall taste function. This potentially explains why there is much conflicting data pertaining to taste function/sensitivity and its link with dietary intake.

It was hypothesized that those who were able to detect and recognize a stimulus at a lower concentration (more sensitive) would consequently perceive a greater intensity when presented suprathreshold concentrations of the same stimulus. This was only observed between the recognition threshold and intensity measures of MSG (umami). None of the detection thresholds were correlated to the perceived intensities within the five prototypical tastes. This implies that a low threshold does not necessarily lead to a greater sensation from suprathreshold concentrations. Notably, earlier research has also failed to find relationships between detection thresholds and suprathreshold intensity ratings (Bartoshuk 1978; Mattes 1985; Mojet et al. 2005; Pangborn and Pecore 1982).

Here, those with more FP did not perceive greater intensity from suprathreshold stimuli (including PROP), nor were they more likely to identify taste thresholds at lower concentrations. In accordance, three recent reports did also not find a link between FP number and taste function (Garneau et al. 2014; Feeney and Hayes 2014; Fischer et al. 2013). FP number is a measure of structural anatomy in the periphery and does not account for other factors that may influence taste intensity, such as central gain (Green and George 2004). A limitation of this study is that taste nerve damage caused by for example head trauma or otitis media (Peracchio et al. 2012) was not inquired in this study. Taste nerve damage affects (regional) taste perception without affecting FP density. It has been suggested that a major difference between studies that do or do not find a relationship between FP and taste function is whether or not to exclude individuals with a history or evidence for oral pathology.

Threshold estimates are highly sensitive to the choice of the method. The method used here was based on the ISO standard ascending method of limits; notably, this rapid method takes substantially less time than the adaptive staircase method. However, this speed potentially comes at the cost of reliability. However, other studies using the staircase method have also failed to find relationships between threshold and suprathreshold intensities (Bartoshuk 1978; Mattes 1985). The present study shows correlations between detection and recognition thresholds. Both thresholds indicate taste acuity at low concentrations, which makes a relationship plausible. However, determination of both the detection and recognition threshold with the same method in one session may have potentially biased subjects, leading to stronger correlations than might be obtained compared with testing in separate tasks. A weak correlation was found for sour and no correlation for salt. This is in accordance with a recent study that showed no relationships between detection and recognition threshold for salt and sour established by different methods (Wise and Breslin 2013).

There is need for an innovative method to assess global taste function in order to gain insight to relationships between chemosensation and ingestive behavior and chronic diet. One potential way to classify individuals would be to investigate taste responses to a wide range of stimuli. Increased or decreased taste response to multiple stimuli has recently been referred to as hypergeusia and hypogeusia, respectively (Hayes and Keast 2011). This new terminology is a refinement from the previous de-facto term “supertaster,” whose actual origin was PROP specific. Thus, the notion that individuals are capable of being broadly sensitive or insensitive to a range of tastants and other chemosensory stimuli may provide a way forward when attempting to identify associations between individual taste function, food choice, and diet.

This study has several limitations that may have influenced the lack of relationships between independent measures of taste function. Many compounds can be used to elicit prototypical taste qualities, and this study chose single exemplars to represent each taste quality. It therefore cannot be discounted that different results may have been produced had alternative reference stimuli been used. Last, the population of the study was limited to female students from the [redacted for blind review], who were mostly young adults with a normal BMI.

In conclusion, this pilot study of taste functionality was the first to explore taste thresholds and suprathreshold intensity of the five prototypical taste qualities, quantification of fungiform papillae on the anterior tongue and also evaluate PROP bitterness in a single cohort of women. Overall, present data highlights that there are multiple facets of taste function, and no one measure is able to capture the totality of the sense of taste. More research is needed to develop new methods of taste function assessment, especially when attempting to link taste responses to dietary intake and food choices.
